# Effects of Mindfulness Exercise Guided by a Smartphone App on Negative Emotions and Stress in Non-Clinical Populations: A Systematic Review and Meta-Analysis

**DOI:** 10.3389/fpubh.2021.773296

**Published:** 2022-01-25

**Authors:** Jinlong Wu, Yudan Ma, Yifan Zuo, Kangyong Zheng, Zhenhui Zhou, Yifan Qin, Zhanbing Ren

**Affiliations:** ^1^Department of Physical Education, Shenzhen University, Shenzhen, China; ^2^Institute of Textiles and Clothing, The Hong Kong Polytechnic University, Hong Kong, China; ^3^College of Teaching Common Courses, Shanwei Polytechnic, Shanwei, China; ^4^Department of Sport Rehabilitation, Shanghai University of Sport, Shanghai, China; ^5^Shenzhen Children's Hospital, Shenzhen, China

**Keywords:** smartphone, app, mindfulness, negative emotions, anxiety, depression, stress

## Abstract

**Background:**

Studies have acknowledged that mindfulness exercise guided by a smartphone app has a positive impact on mental health and physical health. However, mindfulness guided by a smartphone app on mental health is still in its infancy stage. Therefore, we conducted a meta-analysis evaluating the effect of mindfulness intervention guided by a smartphone app on negative emotions and stress in a non-clinical population with emotional symptoms.

**Methods:**

We searched major databases, namely, Web of Science, PubMed, Scopus, China National Knowledge Infrastructure (CNKI), and Wanfang, to identify all of the relevant studies published in English or Chinese from their inception until November 9, 2021. The methodological quality of the included studies was assessed with Cochrane risk-of-bias bias assessment tool. Two researchers independently conducted document retrieval, study selection, data extraction, and methodological quality evaluation.

**Result:**

A total of eight studies were included in the study, with 574 subjects (experimental group: 348; control group: 226). A random effects model was selected to combine effect sizes. The results of the meta-analysis showed that mindfulness exercise guided by a smartphone app reduced negative emotions [standardized mean difference (SMD) = −0.232, 95% CI: −0.398 to −0.066, *p* = 0.006], depressive symptoms (SMD = −0.367, 95% CI: −0.596 to −0.137, *p* = 0.002), and anxiety symptoms (SMD = −0.490, 95% CI: −0.908 to −0.071, *p* = 0.022).

**Conclusions:**

The findings indicate the potentially beneficial effect of mindfulness exercise guided by a smartphone app on symptoms of depression and anxiety among individuals in a non-clinical population with emotional symptoms. Considering the small number and overall methodological weakness of the included studies and lack of randomized controlled trials (RCTs), the results should be interpreted with caution, and future rigorously designed RCTs are warranted to provide more reliable evidence.

## Introduction

The WHO reported that about 8% of the world's population has an emotional disorder (e.g., depression or anxiety), which is one of the leading causes of disability and mortality (1). Patients with emotional disorders often have adverse psychological and physiological effects at the same time, which not only threaten personal health and reduce the quality of life, but also burden families and society (2, 3). At present, the main treatment methods for patients with emotional disorders are psychotherapy and drug therapy. However, clinical drug treatment causes different side effects in patients, along with a high cost of treatment and limited treatment effectiveness (2). Research has shown that, through the development of appropriate mental health projects and plans, early prevention and screening can effectively prevent these mental health problems (2). However, if emotional symptoms are not regulated through intervention, there will be even more negative emotional consequences or they can develop into depression, anxiety, and other emotional disorders (4, 5). Hence, prevention (instead of intervention) among the non-clinical population with emotional symptoms is of great importance.

Mindfulness is an awareness that emerges through a conscious, present, and uncritical way of focusing on the experience that is present from moment to moment (6, 7). Its benefits include enhancing consciousness, strengthening self-regulation, strengthening openness and acceptance of current experience, and looking at problems from different perspectives (6, 7). At present, mindfulness-based interventions include the Buddhist tradition of Vipassana and Zen, which are now standardized group meditation exercises (e.g., mindfulness decompression and mindfulness-based cognitive therapy), and mindfulness-based psychological intervention [e.g., dialectical behavior therapy and Acceptance and Commitment Therapy (ACT)] (8). A large number of studies have confirmed that mindfulness therapy has a strong psychological impact on clinical and non-clinical populations, such as increased attention, reduced stress, less anxiety, and fewer negative emotions (9–12). Therefore, mindfulness therapy is an effective intervention for traditional medicine and psychotherapy globally (2). However, traditional mindfulness exercise takes a long time, learning costs are high, and teachers are relatively scarce. Moreover, effective mindfulness delivery media have been shown not only to assist in face-to-face mindfulness intervention but also to help those who cannot actively seek professional help or are prone to discontinue treatment for some reason (13, 14).

With the continuous development of health-related smartphone apps or smartphones, this emerging field is increasingly becoming a trend. People regularly use smartphones to acquire health knowledge or seek more healthy methods of living from the smartphone, which has enabled people to think about whether it is possible to combine apps in smartphones with psychological research (8). At present, intervention measures based on smartphone apps may further break the space-time limitation of training, improve training motivation, and broaden application prospects (15). Mani et al. (14) evaluated 23 apps in the apple app store found some smartphone apps that specialize in mindfulness intervention have a sharing function or provide access to an app community directly within the app, which can help users share and discuss their mindfulness experiences and challenges in routine exercises, motivate users to participate in health activities, and improve users' mental health level and wellbeing (14). Moreover, the unlimited time and place characteristics of these apps can meet the needs of modern people, which can not only assist in face-to-face mindfulness intervention but can also help those who are unable to seek professional help actively or who are prone to discontinue treatment for some reason, especially young people who enjoy their smartphones but lack the willingness to actively ask for help. Thus, choosing as smartphone app for mindfulness training is the right choice for many people. Studies have shown that electronic health intervention based on mindfulness and relaxation has a positive impact on the physical and mental health of patients with diseases (16). Another study found that mindfulness exercise guided by a smartphone app had a positive effect on relaxing and distracting children with chronic diseases (17). In addition, mindfulness intervention based on network and smartphone device media has been proven effective in reducing stress (18). The above findings indicate that mindfulness-guided exercise by a smartphone app has a positive impact on mental health and physical health.

Therefore, the primary purpose of this study was to analyze the effectiveness of mindfulness exercise guided by a smartphone app on negative emotions and stress in a non-clinical population to provide evidence of mindfulness exercise guided by a smartphone app on mental health.

## Methods

### Search Strategy

The procedure for this systematic review adopted the Preferred Reporting Items for Systematic Review and Meta-Analysis Protocols (PRISMA-P) (18). We used a wide range of search strategies. We searched three English databases (PubMed, Web of Science, and Scopus) and two Chinese databases (China National Knowledge Infrastructure (CNKI) and Wanfang). The search time frame was from the establishment of the database to November 9, 2021, and there was no language restriction. To maximize relevant research, we used three sets of keywords: (1) “smartphone” OR “App” OR “Application” OR “mobile”; (2) “mindfulness” OR “meditation”; and (3) “affective” OR “mood” OR “emotion” OR “depression” OR “anxiety ”OR “stress.” The PubMed search strategy is provided as an example (Supplementary Appendix 1). In addition, the reference lists of eligible studies were manually searched to ensure that all meta-analysis studies were included as much as possible.

### Inclusion Criteria and Study Selection

First, two researchers independently screened based on the title and abstract. They considered studies involving the impact of a mindfulness intervention on a non-clinical population with emotional symptoms using smartphone apps, eliminating repetitive, irrelevant, and review literature, and determining the full-text literature. Studies could be included in this review only when they met the following inclusion criteria: (1) experimental research published in peer-reviewed journals; (2) participants had no physical and/or emotional disorders related diseases; (3) any intervention research on mindfulness exercise guided by a smartphone app as an intervention means (mindfulness intervention only) or as a major component but not the only component of a therapy (integrated mindfulness intervention), and the mindfulness intervention part of the smartphone apps being mainly based on self-guided software rather than by a therapist; (4) at least one outcome measure associated with negative emotions (depression and/or anxiety) or stress; and (5) available quantitative data for effect size calculation. Studies that did not meet the above inclusion criteria were excluded. If there was any discrepancy between the two researchers, a third researcher was consulted until a consensus was reached.

### Descriptive Data Extraction

Descriptive data were extracted independently by two researchers after reading the full text that includes the following four parts: (1) participants (i.e., age, the proportion of women, and sample size); (2) intervention plan (i.e., weekly dose and total time); (3) results (i.e., depression, anxiety, or/and stress) and tools; and (4) to study the beneficial effects of a mindfulness intervention on negative emotions in smartphone apps, we also extracted quantitative data (on depression, anxiety, or/and stress). If there were two tools to measure depression, we extracted both tools and data.

### Assessment of Risk of Bias

The risk of bias was assessed using the Cochrane risk-of-bias bias assessment tool (19). This measure comprises six areas of the trial design: sequence generation, allocation concealment, blinding of assessors, incomplete outcome data, selective outcome reporting, and other sources of bias. Items were rated as high risk, low risk, or unclear risk of bias. Disagreements about the risk of bias assessments were resolved by consensus or by consulting the third author.

### Data Analysis

To extract data accurately, one researcher extracted the data, while another researcher confirmed the extracted data to ensure the accuracy of the data. In this study, Comprehensive Meta-analysis Version 2 was used to carry out the combined effect size after data extraction, and all studies in analysis share a common true effect, we choose fix-effects to calculate effect sizes, if not, we choose the random-effects model (20).The standard mean difference (SMD) was selected as the effect scale index for statistics. The effect size indicated the degree of influence of mindfulness therapy guided by the smartphone app on the negative emotions of a non-clinical population with emotional symptoms (20). The size of the collective effect was calculated (standardized mean difference: negligible effect: 0–0.19; small effect: 0.2–0.49; moderate effect: 0.5–0.79; large effect: >0.8) (20). Furthermore, *I*^2^ was used to evaluate the heterogeneity of the selected studies. The larger the *I*^2^ statistic, the greater the heterogeneity; the low-, medium-, and a high degrees of heterogeneity were expressed by *I*^2^ statistics of 25, 50, and 75%, respectively (20). Funnel plot and Egger tests were used to assess publication bias in more than five studies (21). The “leave-one-out” method was used for sensitivity analysis to determine the source of its heterogeneity (20). For studies that did not provide the above data, the author was contacted by email to obtain the data to improve the quality of this study.

## Results

### Literature Search

**Figure 1:**
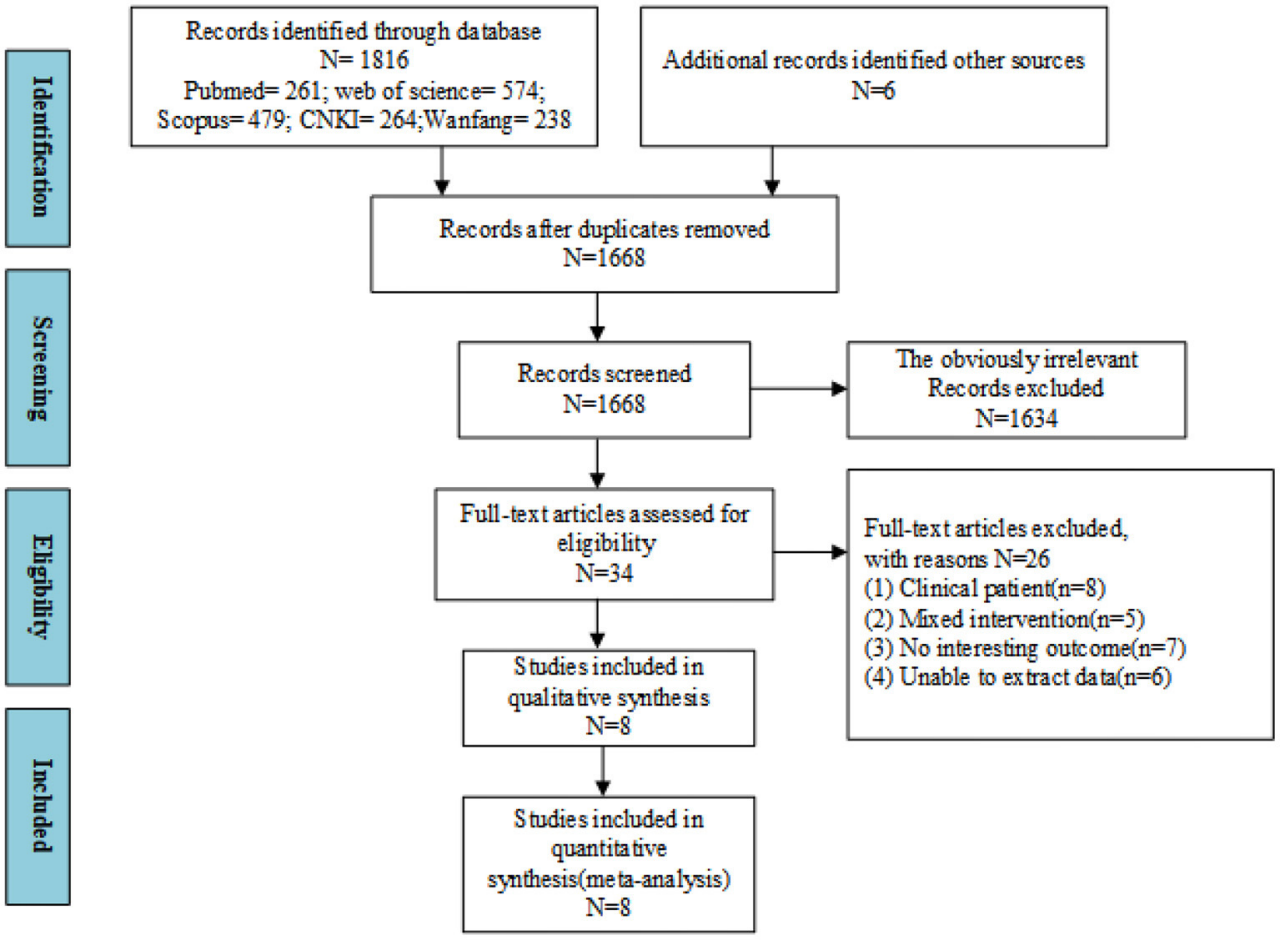
The detailed process of trial selection.

In this study, a total of 1,822 records were retrieved through an electronic database and manual search. First, the search records were checked for duplication, and a total of 154 duplicate records were deleted. The remaining 1,668 records were further screened according to the study signs (title and abstract), and 1,634 records that did not meet the criteria of this study were deleted. Finally, 34 studies were evaluated for full-text qualification, and 26 studies were found not to meet the predetermined inclusion criteria. Finally, eight studies were identified for meta-analysis. The flow chart of the literature search and research selection is shown in Figure 1.

### Research Characteristics

All of the research characteristics are summarized in Table 1. We summarized eight studies on the impact of mindfulness intervention in the smartphone app on a non-clinical population with emotional symptoms. These studies were published from 2015 to 2020; among the eight studies, one (22) was conducted in Italy, four (23–26) were conducted in the United States, one (27) was conducted in the United Kingdom, one (28) was conducted in South Korea, and one (28) was an experiment conducted in China. A total of 574 people were enrolled (experimental group: 348; control group: 226). The subjects were mainly workers (22), hospital staff (23, 24, 28, 29), students (25, 26), and company staffs (27). Six studies (22, 24, 25, 27–29) compared the mindfulness exercise guided by a smartphone app group with a control group, and two studies (23, 26) only researched mindfulness exercise guided by a smartphone app group. There were individual differences in the intervention time, ranging from 70 to 420 min per week and duration ranging from 3 to 8 weeks. Moreover, two studies (22–24) measured the overall negative emotion variables, five studies (24, 26–29) measured depression variables, five studies (24, 26–29) measured anxiety variables, and three (24–26, 28) studies measured stress variables.

**Table 1:** Characteristics of eligible studies.

**References**	**Study design**	**Study area**	**Profession**	**Sample size (*N*)**	**Female (%)**	**Mean age (years)**	**Type of APPs**	**Weekly dosage**	**Duration**	**Emotion outcomes**
Carissoli et al. (22)	Randomized controlled design	Italy	Workers	EG: 20 CG: 18	Total: 84.21%	Total: 38.11 ± 6.92	Designed by the researchers	210 min	3 weeks	Depression, anxiety: MSP
Wen et al. (23)	Prospective, pilot design	USA	Hospital staffs	EG: 30	EG: 60%	NR	Freely download (head space)	More than 70 min	4 weeks	Negative emotions: NAS
Mistretta et al. (24)	Randomized controlled design	USA	Hospital staffs	EG: 23 CG: 15	EG: 78.3% CG: 93.3%	EG: 43.7 ± 14.7 CG: 46.1 ± 10.5	Designed by the researchers	120 min	6 weeks	Depression: DASS-Depression Anxiety: DASS-Anxiety Stress: DASS-Stress
Yang et al. (25)	Prospective, randomized controlled design	USA	Medical students	EG: 44 CG: 44	Total: 64	NR	Freely download (head space)	70–140 min	30 days	Stress: PSS
Moffitt-Carney et al. (26)	Pretest–posttest design	USA	College students	EG: 59	EG: 74.6	EG: 19.92 ± 3.87	Pay to download	420 min	5 weeks	Depression: CESD-R Anxiety: STAI
Bostock et al. (27)	Randomized controlled design	UK	Company staffs	EG: 108 CG: 81	EG: 60 CG: 58	EG: 36 ± 8.3 CG: 35 ± 6.9	Freely download (head space)	70–140 min	8 weeks	Depression: HADS-Depression Anxiety: HADS-Anxiety
Hwang et al. (28)	Randomized controlled design	South Korea	Nurses	EG: 26 CG: 30	EG: 83.3 CG: 93.3	<30 years old: 14 31–40: 30 >40 years old: 12	Designed by the researchers	More than 70 min	4 weeks	Depression: PHQ-9 Anxiety: GAD-7 STRESS: PSS-10; KOSS
Wu et al. (29)	Randomized controlled design	China	Nurses	EG: 38 CG: 38	EG: 100 CG: 100	EG: 29.84 ± 4.66 CG: 31.92 ± 4.82	Freely download	140–420 min	8 weeks	Depression:SCL-Depression Anxiety: SCL-Anxiety

*EG, Experimental Group; CG, Control Group; NR, Not Reported; MSP, The Mesure du Stress Psychologique; NAS, The PANAS [Positive Affect Scale [PAS] and Negative Affect Scale]; DASS-21, The Depression, Anxiety, and Stress Scales; PSS, Perceived Stress Scale; CESD-R, The Center for Epidemiologic Studies Depression Scale Revised; STAI, The State-Trait Anxiety Inventory; HADS, The Hospital Anxiety and Depression Scale; PHQ-9, The Patient Health Questionnaire; GAD-7, The Generalized Anxiety Disorder; PSS-10, The assessed self-awareness of stress; KOSS, The Korean Occupational Stress Scale; SCL-90, Self-reporting inventor*.

### Risk of Bias

**Figure 2:**
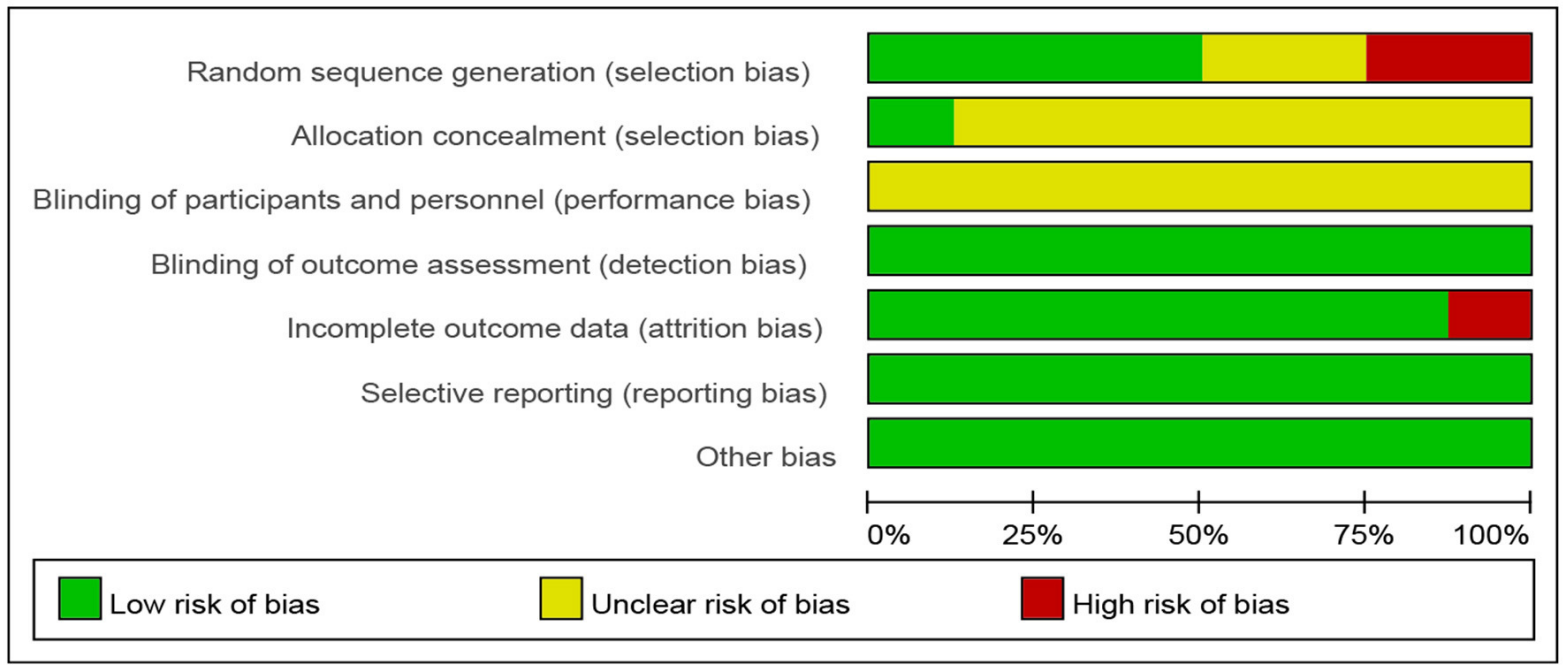
Risk of bias graph: each risk of bias item is presented as percentages.

**Figure 3:**
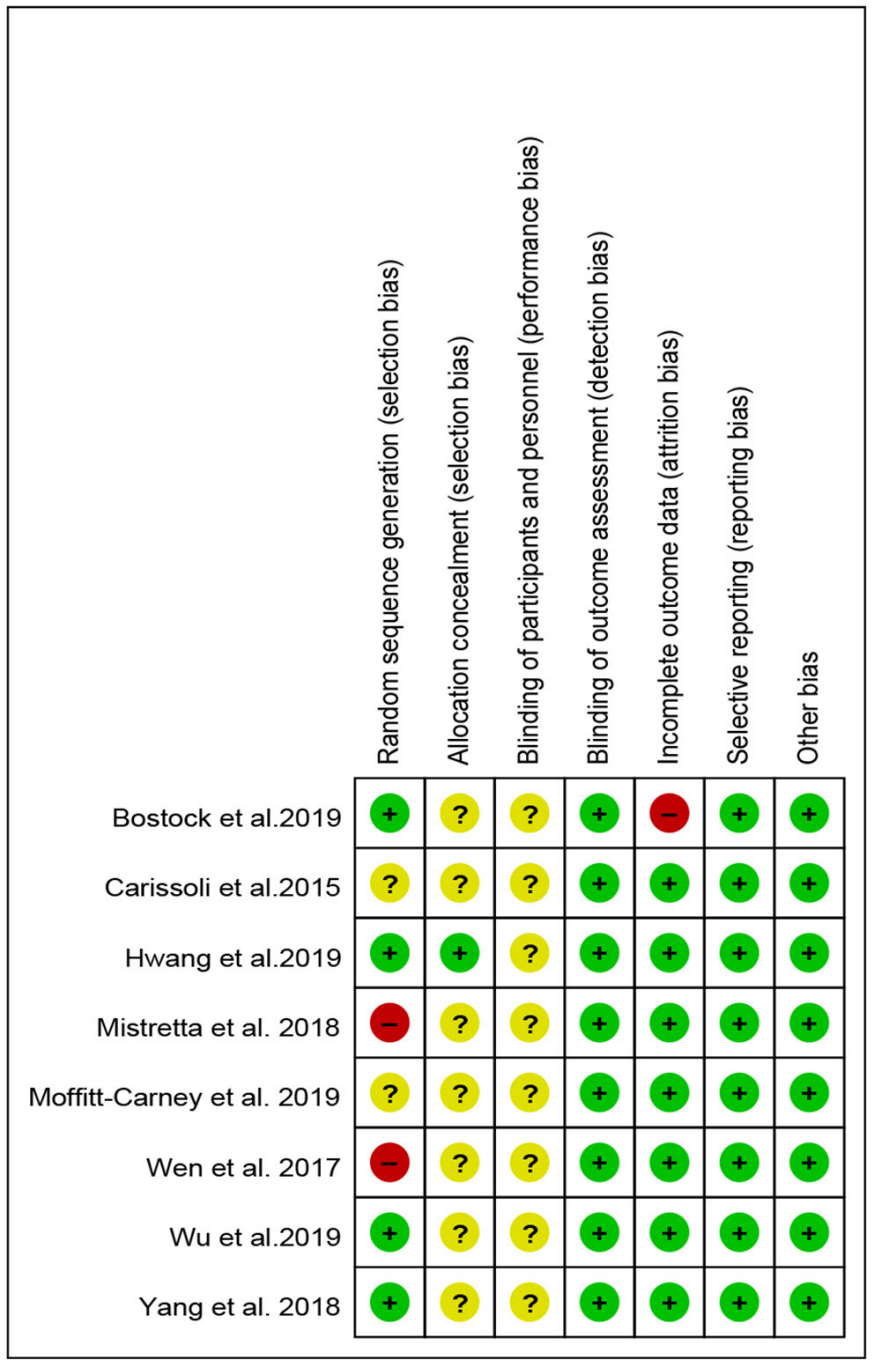
Risk of bias of included studies. Green: low risk of bias; yellow: unclear risk of bias; red: high risk of bias.

Across studies, the risk of bias for blinding, incomplete outcome data, selective reporting, and other biases was sufficient in >80% of the studies. Random sequence generation was sufficient in >50% of the studies. One study (12.5%) sufficiently allocated concealment. The risk of bias across studies is reported in Figure 2. Furthermore, the risk of bias per study at the item level is shown in Figure 3.

### Effect of Mindfulness Exercise Guided by a Smartphone App on Negative Emotions

**Figure 4:**
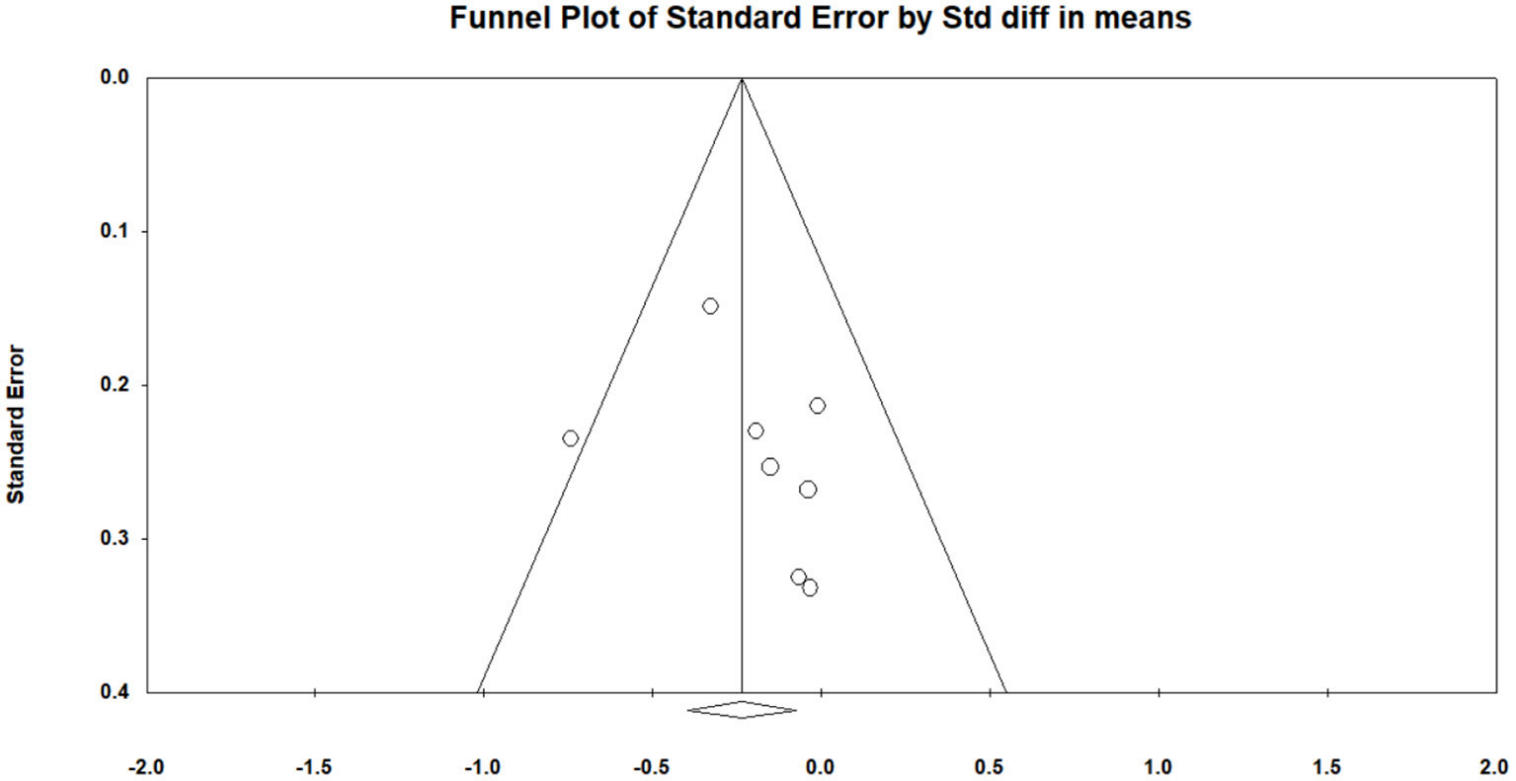
Funnel plot for negative emotions.

**Figure 5:**
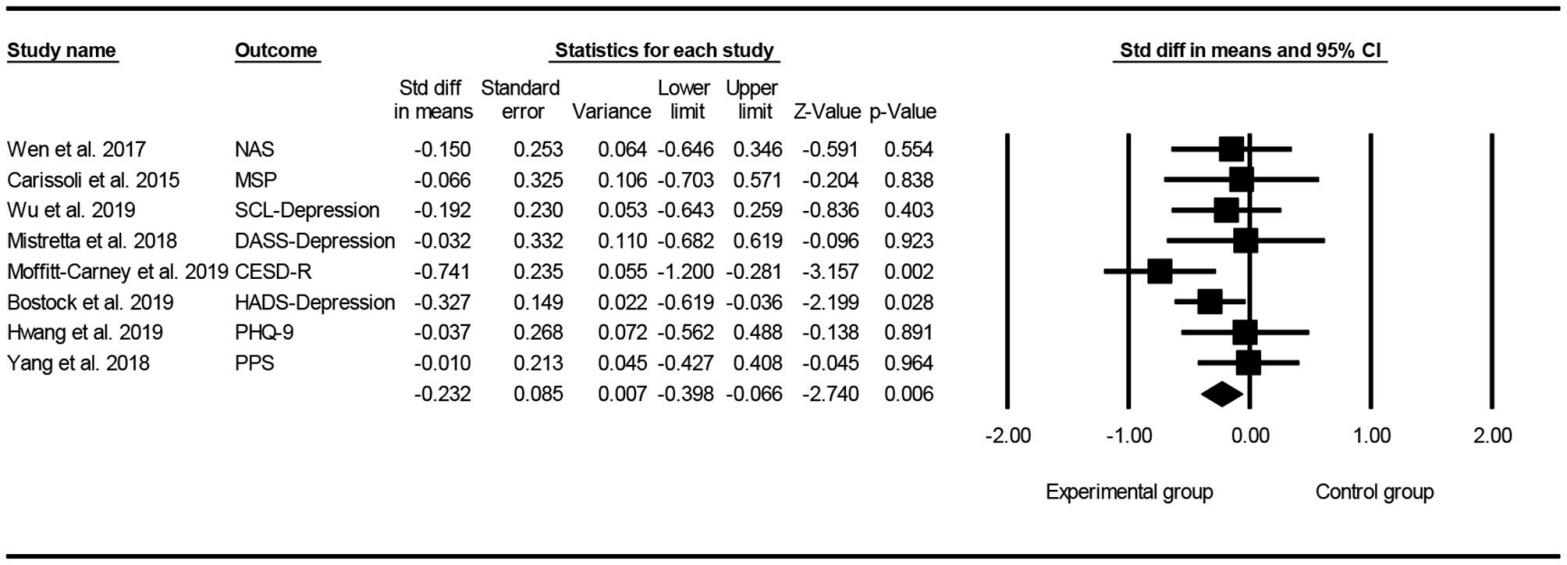
Forest plot for negative emotions.

All of the selected studies were included in a random model to analyze the effect of mindfulness intervention in the smartphone app on negative emotions (depression and anxiety). Conservatively, if a study contained more than one negative emotion, the tiniest change score of negative emotion was selected for analysis. The funnel diagram is obtained by combining the random effect model, as shown in Figure 4 (Egger's regression intercept = 1.370, *p* = 0.389). The meta-analysis of the random model showed that mindfulness exercise guided by a smartphone app had small effects on negative emotions (SMD = −0.232, 95% CI: −0.398 to −0.066, *I*^2^ = 6.503%, *p* < 0.01, Figure 5). The sensitivity analysis of “leave-one-out” was subsequently carried out (SMD = −0250 to −0.167).

### Effect of Mindfulness Exercise Guided by a Smartphone App on Depression

**Figure 6:**
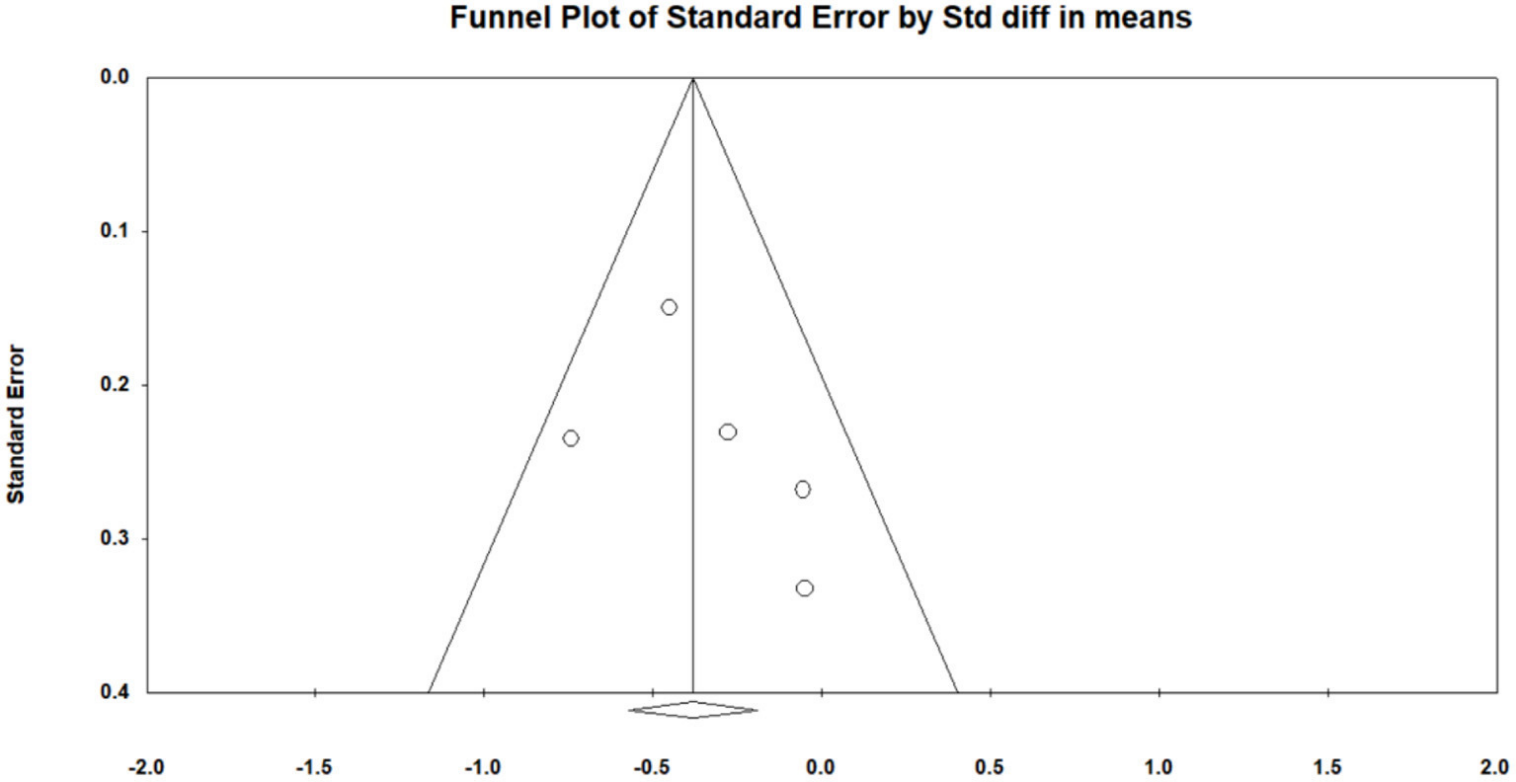
Funnel plot for depression.

**Figure 7:**
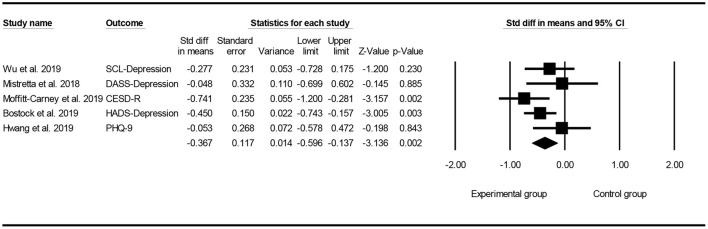
Forest plot for depression.

Of the seven included studies, five studies (24, 26–29) used depression as the main outcome, and a meta-analysis was conducted to test the overall effect of mindfulness intervention in a smartphone app. Owing to the difference in measurement results, a random effect analysis was performed to combine the results, and the funnel map of depression was obtained, as shown in Figure 6 (Egger's regression intercept = 1.925, *p* = 0.385). The meta-analysis showed that mindfulness exercise guided by a smartphone app had small effects on depression (SMD = −0.367, 95% CI: −0.596 to −0.137, *I*^2^ = 24.091%, *p* < 0.01 0.002, Figure 7). Subsequently, the sensitivity analysis of “leave-one-out” was carried out (SMD = −0.427 to −0.306).

### Effect of Mindfulness Exercise Guided by a Smartphone App on Anxiety

**Figure 8:**
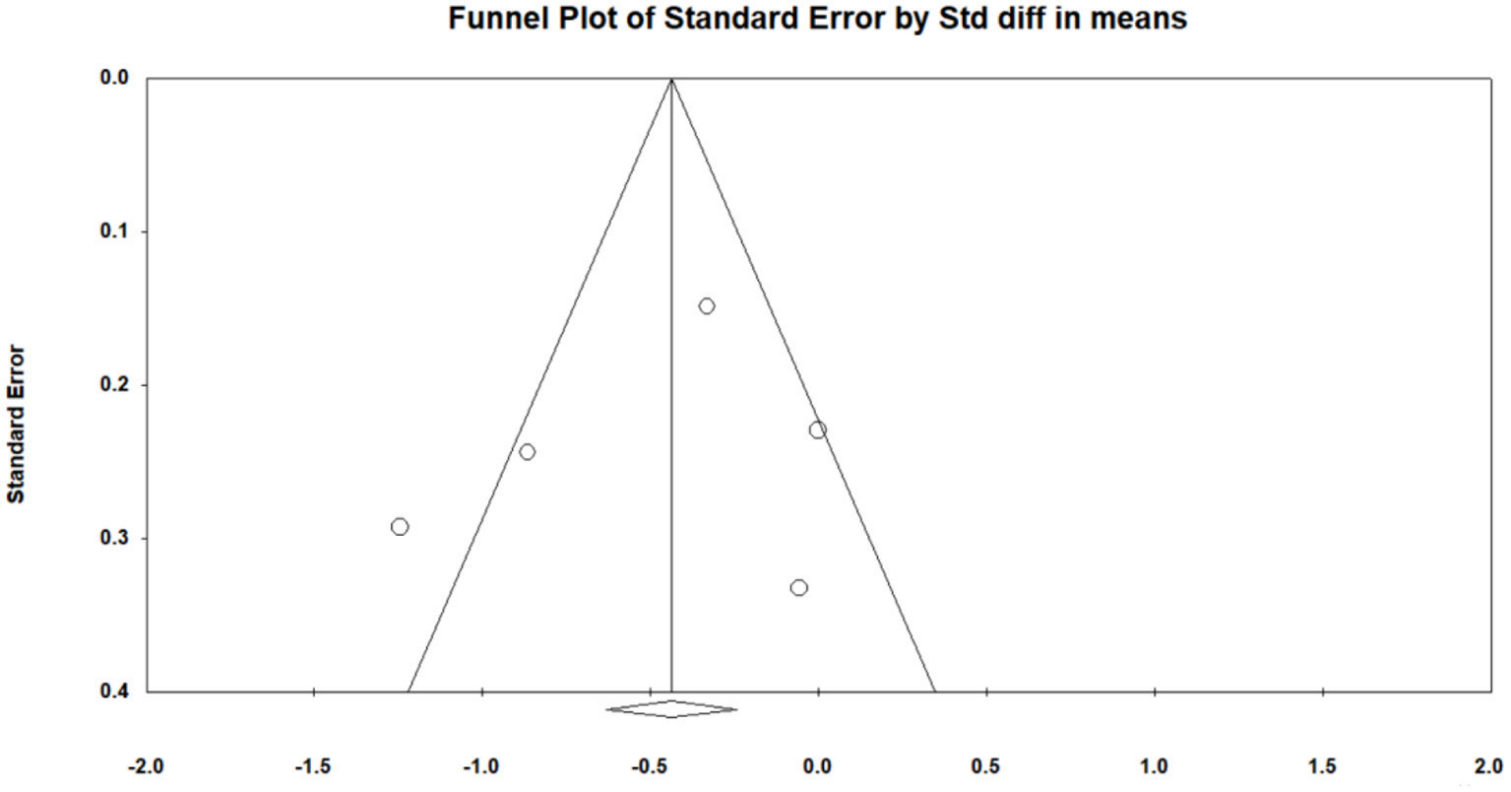
Funnel plot for anxiety.

**Figure 9:**
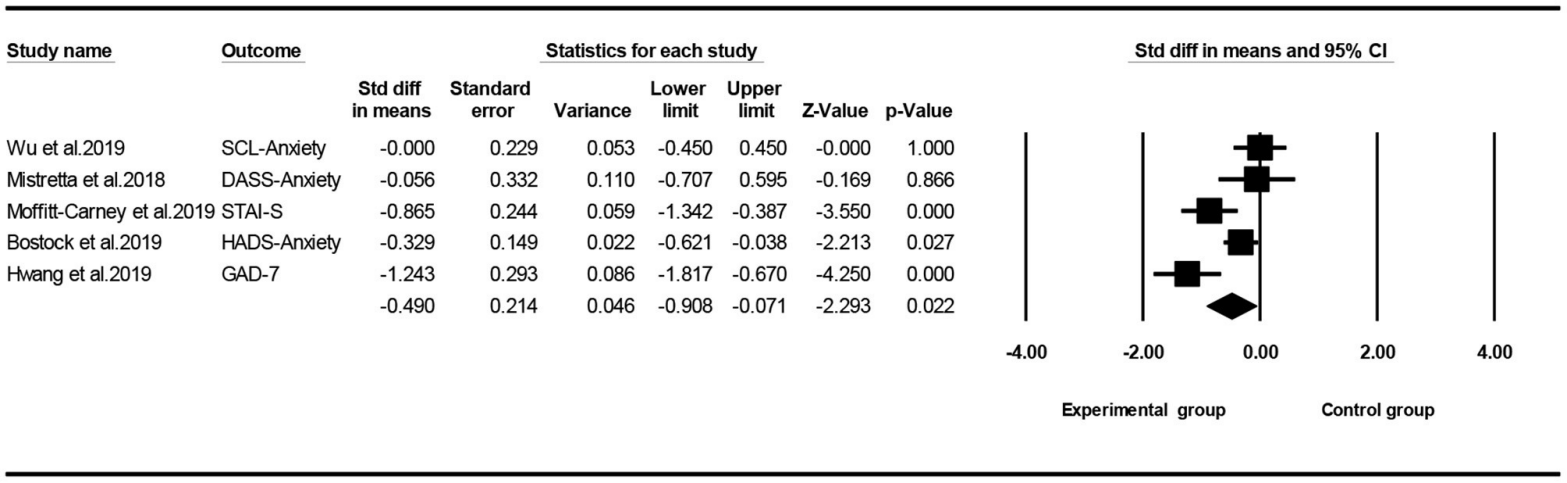
Forest plot for anxiety.

Of the seven included studies, five studies (24, 26–29) used anxiety as the main outcome, and a meta-analysis was conducted to test the overall effect of mindfulness intervention in a smartphone app. Owing to the difference in the outcome measurement, a random effect analysis was performed to combine the results, and the funnel map of anxiety was obtained, as shown in Figure 8 (Egger's regression intercept = −1.795, *p* = 0.641). The meta-analysis showed that, compared with the control group, the mindfulness exercise guided by a smartphone app had small effects on anxiety (SMD = −0.490, 95% CI: −0.908 to −0.071, *I*^2^ = 75.233%, *p* < 0.05, Figure 9). Subsequently, the sensitivity analysis of “leave-one-out” was carried out (SMD = −0.619 to −0.327).

### Effect of Mindfulness Exercise Guided by a Smartphone App on Stress

**Figure 10:**
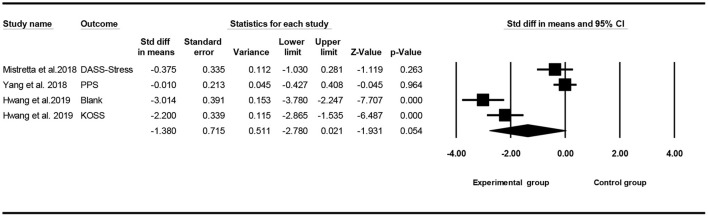
Forest plot for stress.

Of the seven included studies, four studies (24–26, 28) considered the stress variable as the main outcome, and a meta-analysis was conducted to test the overall effect of mindfulness exercise guided by a smartphone app. Owing to the difference in the outcome measurement, a random effect analysis was performed to combine the results. The meta-analysis showed that, compared with the control group, mindfulness exercise guided by a smartphone app had no effect on stress (SMD = −1.380, 95% CI: −2.780 to 0.021, *I*^2^ = 95.299%, *p* > 0.05, Figure 10).

## Discussion

This study involved a meta-analysis evaluation of the effects of mindfulness intervention guided by a smartphone app on negative emotions (depression and anxiety) and stress on a non-clinical population with emotional symptoms. Specifically, our study showed that mindfulness intervention guided by a smartphone app had a small-to-medium improvement effect on the negative emotions of the non-clinical population with emotional symptoms. The meta-analysis results were as follows: negative emotions (SMD = −0.272), depressive symptoms (SMD = −0.367), and anxiety symptoms (SMD = −0.490). The novel findings based on available evidence indicate that a favorable effect of mindfulness exercise guided by a smartphone app on reducing anxiety was found in the treated group compared to the untreated group. However, more studies with high homogeneity in methodology are needed to confirm these findings. The details of the study follow.

We included items of negative emotion data, four items of depression data (24, 26, 28, 29), and one item of stress data (25) from two studies (22, 23) to analyze the effect of mindfulness exercise guided by a smartphone app on negative emotions. The subjects included were mainly workers, students, nurses, and hospital staff. We speculate that the reason the researchers chose this kind of population is that the experimental subjects were relatively concentrated. Because of the particularity of each work or study, there was a higher chance of reducing negative emotions, especially medical workers. Our meta-analysis found that practitioners who practiced mindfulness exercises guided by a smartphone app exhibited a moderate effect of reduced negative emotions. This result was encouraging and indicates that mindfulness exercise guided by a smartphone app is effective in reducing negative emotions. Similarly, depression and anxiety calculations gave very similar results, both were also believed to be reduced through mindfulness exercise guided by a smartphone app. These benefits from the mindfulness exercise guided by a smartphone app to select text, audio, or video may be provided in these apps to explain mindfulness, which can help users practice mindfulness step by step and can provide long-term, detailed, and instructive mindfulness education for practitioners. Wen et al. found that the generation of negative emotions of medical workers was related not only to stress but also a factor of negative emotions in high-intensity medical work and that practicing mindfulness could help reduce the degree of fatigue of medical workers (23). Carissoli et al. pointed out that the mindfulness intervention time for the mindfulness exercise guided by a smartphone app should be more than 3 weeks to ensure that the practitioners have confidence (22). However, Bostock et al. found that 4 weeks of intervention exercises were not enough. To improve the benefits of mindfulness exercise, and thereby improve depression and anxiety, the development of mindfulness apps is necessary for the future (27).

In terms of reduced stress, we included a total of four studies. The meta-analysis results showed that the *p* was 0.054, which was higher than 0.05. Besides, the result had high heterogeneity, which led to this result. Potential sources of heterogeneity may include different tools to measure stress from four studies. After detailed analysis and interpretation of each study, Mistretta et al. found that there were significant differences in the stress variables of the mindfulness intervention group guided by the smartphone app, and studies have shown that such interventions are limited in reducing stress (24). A study by Hwang et al. found that mindfulness exercise guided by a smartphone app not only reduced nurses' feelings of pressure but also improved their self-efficacy (28). Therefore, we have reason to believe that mindfulness exercise guided by a smartphone app is a promising method for reducing personal stress.

Although mindfulness intervention with a smartphone app has certain advantages, the quality and intervention effect of many apps still needs to be explored. We speculate that there may be certain reasons why mindfulness exercise guided by a smartphone app has a small effect on students' depression and anxiety: (1) some apps may not thoroughly explain the concept of mindfulness, and some apps may not necessarily be related to mindfulness or meditation in terms of their functions or features. There is still no perfect standard for the concept of mindfulness and the steps of mindfulness training. (2) The functions of smartphone apps related to mindfulness training are limited, and most of them are aimed at short-term individual practices. There is no app for mindfulness professionals or those who want to carry out mindfulness training for a long time. Moreover, there is no possibility of group education or the practice of mindfulness. Therefore, although the smartphone app is a useful auxiliary medium for mindfulness intervention, its potential still needs to be explored to a large extent while improving its existing functions.

## Study Limitations

There were limitations to this study. First, the study data were retrieved in November 9, 2021, and the findings of possible related studies (ongoing studies, manuscripts under review, and studies published after that date) were excluded. Second, we only investigated the effect of mindfulness exercise guided by a smartphone app on the negative emotions and stress of a non-clinical population with emotional symptoms, but we did not investigate neural mechanism-related research. Third, only eight studies were included in this study; these relatively small number of studies translated to less overall real data, which may have affected the quality of research. Fourth, owing to the limited number of studies included, it was difficult to conduct subgroup analysis on intervention time and long-term effect after the intervention.

## Conclusion

The results indicate that mindfulness exercise guided by a smartphone app shows potential advantages in reducing depression and anxiety in a non-clinical population with emotional symptoms. This suggests that mindfulness exercise guided by a smartphone app is of great significance in preventing emotional disorders and promoting mental health.

## Data Availability Statement

The original contributions presented in the study are included in the article/Supplementary Material, further inquiries can be directed to the corresponding author/s.

## Author Contributions

JW, YM, and ZR contributed to the conception and design of the review. JW and YZ applied the search strategy. JW and KZ applied the selection criteria. ZZ and ZR completed an assessment of the risk of bias. YM analyzed and interpreted the data. JW and YM wrote this manuscript. YQ edited this manuscript. ZR is responsible for the overall project. All authors have read and approved the manuscript.

## Funding

Research Foundation for Young Teacher of Shenzhen University (grant number QNJS0274); High-Level Scientific Research Foundation for the Introduction of Talent of Shenzhen University (grant number RC00228); High-Level Scientific Research Foundation for the Introduction of Talent of Shanwei Institute of Technology (grant number SKQD2021B-028); and the Natural Science Featured Innovation Projects in Ordinary Universities in Guangdong Province (grant number 2021KTSCX297).

## Conflict of Interest

The authors declare that the research was conducted in the absence of any commercial or financial relationships that could be construed as a potential conflict of interest. The reviewer SL declared a shared affiliation with one of the authors, KZ, to the handling editor at the time of review.

## Publisher's Note

All claims expressed in this article are solely those of the authors and do not necessarily represent those of their affiliated organizations, or those of the publisher, the editors and the reviewers. Any product that may be evaluated in this article, or claim that may be made by its manufacturer, is not guaranteed or endorsed by the publisher.
