# Supplementary material for: Effects of Mindfulness Exercise Guided by a Smartphone App on Negative Emotions and Stress in Non-Clinical Populations: A Systematic Review and Meta-Analysis
Source: Front Public Health. 2022 Jan 25;9:773296. doi: 10.3389/fpubh.2021.773296 (PMC8825782; doi:10.3389/fpubh.2021.773296)
Supplement: Supplementary file 1 [file Table_1.DOCX]

**Supplementary materials**

Pubmed

| Search | Query | Items found |
| --- | --- | --- |
| #1 | Search: ((smartphone[Title/Abstract]) OR (App[Title/Abstract])) OR (Application mobile[Title/Abstract]) | 146538 |
| #2 | Search:(mindfulness[Title/Abstract]) OR (meditation[Title/Abstract]) | 13389 |
| #3 | Search:(((((affective[Title/Abstract]) OR (mood[Title/Abstract])) OR (emotion[Title/Abstract])) OR (depression[Title/Abstract])) OR (anxiety[Title/Abstract])) OR (stress[Title/Abstract]) | 1377826 |
| #4 | #1 and #2 and #3 | 261 |
